# *Iberis amara* Extract Induces Intracellular Formation of Reactive Oxygen Species and Inhibits Colon Cancer

**DOI:** 10.1371/journal.pone.0152398

**Published:** 2016-04-06

**Authors:** Christopher Weidner, Morten Rousseau, Annabell Plauth, Sylvia J. Wowro, Cornelius Fischer, Heba Abdel-Aziz, Sascha Sauer

**Affiliations:** 1 Otto Warburg Laboratory, Max Planck Institute for Molecular Genetics, Berlin, Germany; 2 Scientific Department, Steigerwald Arzneimittelwerk GmbH, Darmstadt, Germany; 3 CU Systems Medicine, University of Würzburg, Würzburg, Germany; Duke University Medical Center, UNITED STATES

## Abstract

Massively increasing global incidences of colorectal cancer require efficient treatment and prevention strategies. Here, we report unexpected anticancerogenic effects of hydroethanolic *Iberis amara* extract (IAE), which is known as a widely used phytomedical product for treating gastrointestinal complaints. IAE significantly inhibited the proliferation of HT-29 and T84 colon carcinoma cells with an inhibitory concentration (IC_50_) of 6 and 9 μg/ml, respectively, and further generated inhibitory effects in PC-3 prostate and MCF7 breast cancer cells. Inhibition of proliferation in HT-29 cells was associated with a G2/M phase cell cycle arrest including reduced expression of various regulatory marker proteins. Notably, in HT-29 cells IAE further induced apoptosis by intracellular formation of reactive oxygen species (ROS). Consistent with predictions derived from our *in vitro* experiments, bidaily oral gavage of 50 mg/kg of IAE over 4 weeks resulted in significant inhibition of tumor growth in a mouse HT-29 tumor xenograft model. Taken together, *Iberis amara* extracts could become useful alternatives for preventing and treating the progression of colon cancer.

## Introduction

Colorectal cancer (CRC) is the third most common cancer globally and the fourth leading cause of cancer-related death [1]. CRC accounts for 1–2 million new cases and more than 600,000 deaths per year [1]. The global incidence of CRC is steadily increasing and seems to be associated with a western lifestyle [1].

Efficient therapy of CRC is hampered by reduced compliance with screening recommendations, late stage diagnosis of new CRC cases, severe therapy toxicity, drug resistance, and cancer relapse [2, 3]. To overcome these limitations novel approaches for effective prevention and treatment of CRC are needed, including complementation of strong cytotoxic or targeted drug treatments, for example using safe phytotherapeutic alternatives [4].

In general, pools of natural substances such as flavonoids, phenolic acids, polyphenols and terpenoids can modulate multiple molecular pathways involved in the pathobiology of complex diseases. Notably, plant-based compound mixtures generally provoke low toxic side effects [5]. Recent studies have shown that mild herbal extracts reduce cancer cell growth, by inducing apoptosis and producing synergistic effects in CRC. For example, extracts from green tea, grapes, ginger, turmeric, soy and garlic [4], chamomile [6] or melissa officinalis [7, 8] were reported to exert health-beneficial effects.

*Iberis amara* (also called bitter candytuft) belongs to the family *Brassicaceae*, and is common in Central and Southern Europe [9]. Here, we analyzed a well-validated and approved hydroethanolic extract of *Iberis amara* (IAE), which is routinely applied for gastrointestinal use.

IAE exerted surprisingly strong antiproliferative and apoptotic activity, for example in colon carcinoma cells. These antitumoral effects were caused by intracellular formation of reactive oxygen species (ROS), whereas quenching of ROS by antioxidants protected cancer cells from apoptosis. Notably, application of IAE in a xenograft mouse model resulted in efficient inhibition of tumor growth *in vivo*, providing evidence that IAE could be beneficially applied for prevention and complementary treatment of colon cancer.

## Materials and Methods

### Materials

Chemicals were purchased from the following sources: staurosporine (STN) and irinotecan (IRI) from LKT Laboratories (Biomol), oxaliplatin (OX) from Cayman Chemical (Biomol, Hamburg, Germany). Paclitaxel (PTX), 5-fluorouracil (5-FU), menadione (MD), tert-butyl hydroperoxide (TBHP), N-acetyl cysteine (NAC), glutathione (GSH), 3H-1, 2-dithiole-3-thione (D3T), α-tocopherol (α-TOC) and ascorbic acid (AA) were purchased from Sigma Aldrich (Taufkirchen, Germany). Hydroethanolic *Iberis amara* extract (IAE) is an industrial, validated hydroethanolic (31% v/v) extract provided by Steigerwald Arzneimittelwerk (Darmstadt, Germany). The extract was produced according to a standardized procedure. Quality was controlled by means of HPLC fingerprinting, and the content of the extract was determined exactly as described earlier in full detail [10].

### Cell culture

Human HT-29 (ACC-299, DSMZ, Braunschweig, Germany) and T84 colorectal cancer cells (CCL-248, ATCC, LGC Promochem, Wesel, Germany) were cultured in Dulbecco's Modified Eagle Medium/Nutrient Mixture F-12 (DMEM/F-12, #11330–057, Gibco, Life Technologies, Darmstadt, Germany) supplemented with 5% fetal bovine serum (FBS, Biochrom, Berlin, Germany) and 100 U/ml penicillin and 100 μg/ml streptomycin (Biochrom). HT-29 cells were established from the primary tumour of a 44-year-old Caucasian woman with colon adenocarcinoma; T84 cells were isolated from a lung metastasis of a colon carcinoma in a 72-year-old male. PC-3 prostate cancer cells (CRL-1435, ATCC) were cultivated in RPMI 1640 (Biochrom) supplemented with 10% FBS and 100 U/ml penicillin and 100 μg/ml streptomycin. MCF7 breast cancer cells (HTB-22, ATCC) were cultured in DMEM GlutaMAX (Gibco, Life Technologies) supplemented with 10% FBS, 10 μg/ml human insulin (Sigma Aldrich) and 100 U/ml penicillin and 100 μg/ml streptomycin. CCD 841 CoN primary colon cells (CRL-1790, ATCC) were grown in DMEM GlutaMAX (Life Technologies) containing 10% FBS. All cell lines were maintained at 37°C in a humidified 5% CO_2_ atmosphere. Cells were treated using hydroethanolic IAE, indicated compounds dissolved in DMSO, or corresponding vehicle controls.

Treatments of cells with IAE were in particular optimized in terms of concentration and treatment times, which varied depending on the biological response and markers used for detection.

### Proliferation assay

Proliferation of cancer cells was determined using the CyQUANT NF Cell Proliferation Assay Kit (Life Technologies). For this purpose, HT-29, T8, PC-3 and MCF7 cancer cells, and normal CCD 841 CoN cells, were seeded in black 384-well plates (#3712, Corning, Fisher Scientific, Schwerte, Germany) with a density of 750 cells/well (HT-29), 900 cells/well (T84), 250 cells/well (PC-3), 900 cells/well (MCF7) and 900 cells/well CCD 841 CoN primary colon cells, respectively, in a final volume of 50 μl/well. Cells were then treated with concentration series of compounds by adding 10 μl of a 6-fold stock concentration. After 72 h (HT-29, PC-3) and 96 h (T84, MCF7, CCD 841 CoN) treatment, respectively, the relative number of cells was determined by measurement of cellular DNA content using the fluorescent CyQUANT dye according to the manufacturer’s instructions. For co-treatment with antioxidants, the treatment time was reduced to 48 h. Fluorescence intensity was measured using the POLARstar Omega (BMG Labtech, Ortenberg, Germany) and transformed to relative number of cells. For concentration series, data were fitted using GraphPad Prism 5.0 according to equation: Y = Top + (Bottom-Top)/(1+10^((LogIC_50_-X)*HillSlope)) with variable Hill slope. Efficiency is the maximal observed induction of cell death (100%—Bottom) relative to nontreated cells (set to 0%).

### Cell cycle analysis

Modulation of the cell cycle was determined in HT-29 cells treated with 30 μg/ml IAE for 24 h. After trypsinization cells were fixed in 70% ethanol and incubated on ice for 15 min. Cells were then labeled with propidium iodide (PI)/RNase staining solution (#4087, Cell Signaling Technology, New England Biolabs, Frankfurt, Germany) and further incubated for 15 min at room temperature. Fixed cells were frozen at -20°C before analysis. Finally, cells were analyzed using the FACS Aria II flow cytometer (BD Biosciences, Heidelberg, Germany). Data analysis was performed using FlowJo 7.6 (Tree Star), and cell cycle states were assigned by using the implemented Dean-Jett-Fox model. Histograms were visualized by GraphPad Prism 5.0. Note that in our hands this analysis was unfortunately inefficient when using alternative colon-derived T84 cells due to difficulties to obtain significant numbers of single cells. This problem was even increased during fixation, resulting in accumulating T84 cells (e.g. doublets), which made data analysis of different cell cycle states impossible in practice.

### Immunoblotting

Cells were lysed in 50 mM Tris-HCl (pH 8.0), 10 mM EDTA, 1% SDS with protease inhibitors (Roche Diagnostics) and phosphatase inhibitors (Sigma Aldrich), and sonicated (using a device from Bandelin electronic, Berlin, Germany). After centrifugation at 10,000 g for 10 min, supernatants were stored at -80°C until use. Samples were denaturated and separated by use of NuPAGE Novex 4–12% Bis-Tris Protein gels (Life Technologies) and blotted onto Hybond ECL nitrocellulose membranes (GE Healthcare, Freiburg, Germany). Membranes were blocked with 5% milk powder in 0.1% Triton X-100/TBS (TBS-T, pH 7.6) at room temperature for 1 h and subsequently washed in TBS-T (0.1%). Primary antibodies against cyclin A2 (#4656), cyclin D3 (#2936), CDK 2 (#2546), CDK 4 (#2906), CDK 6 (#3136), caspase 3 (#9665), cleaved caspase 3 (#9664), caspase 9 (#9502), cleaved caspase 9 (#9501), PARP (#9542), cleaved PARP (#5625), p18 (#2596), p21 (#2947, all from Cell Signaling Technology), and GAPDH (#sc-48167, Santa Cruz) were diluted in TBS-T (0.1%) with milk powder and BSA, respectively, according to the manufacturer's protocols. Membranes were shaken at 4°C overnight, subsequently washed with TBS-T (0.1%) and incubated with anti-rabbit IgG-HRP (#sc-2004), anti-mouse IgG-HRP (#sc-2005) and anti-goat IgG-HRP (#sc-2020, all Santa Cruz), respectively, according to the manufacturer's instructions. Proteins were detected using the Western Lightning ECL solution (Perkin Elmer, Rodgau, Germany). Membranes were stripped with Restore Plus Western Blot Stripping Buffer (Thermo Scientific) for 7 min at room temperature. Densitometric analyses were performed using the FUSION-SL Advance 4.2 MP System (Peqlab, Erlangen, Germany).

### Caspase activation

Activation of caspases 2, 3/7, 6, 8 and 9 was investigated using the luminometric Caspase-Glo assays (Promega, Mannheim, Germany). Briefly, one day before treatment HT-29 cells were seeded in black 384-well plates (#3712, Corning) with a density of 2,000 cells/well in a final volume of 20 μl/well. Cells were then treated for 24 h with 60 μg/ml IAE, 10 μM staurosporine or vehicle only. Luminescence was measured according to the manufacturer’s instructions using the POLARstar Omega (BMG Labtech).

### DNA fragmentation assay

BrdU-labeled DNA fragments in the cytoplasm of treated HT-29 cells were quantified using the Cellular DNA Fragmentation ELISA kit (Roche Diagnostics). Briefly, HT-29 cells were seeded in 96-well plates (TPP) with a density of 13,000 cells/well in presence of 10 μM 5-bromo-2'-deoxyuridine (BrdU) and incubated for 2 days at 37°C. Supernatant was removed and the cells were treated for 6 h with IAE, STS or vehicle as indicated in the corresponding results figure. The cytosolic fractions were harvested and analyzed on a 96-well half-area clear high-binding microplate (#3690, Corning). Cell-free samples were used as background control for subtraction, and data were normalized to vehicle-treated cells.

### Phosphatidylserine externalization (annexin V) assay

Externalization of phosphatidylserine was analyzed using the annexin-V-FLUOS Staining kit (Roche Diagnostics, Mannheim, Germany) according to the manufacturer’s instructions. Briefly, HT-29 and T84 cells were treated as indicated in the corresponding results figure and subsequently labeled with Annexin-V-FLUOS and propidium iodide. Flow cytometry was carried out using the Accuri C6 (BD Biosciences, Heidelberg, Germany). Data were analyzed using FlowJo 7.6 (Tree Star). For bar plot presentation, apoptosis comprised early (annexin positive, PI negative) and late stage apoptotic events (annexin positive, PI positive).

### Detection of reactive oxygen species (ROS)

Formation of ROS was detected using the ROS-sensitive CellROX Orange probe (Life Technologies) according to the manufacturer’s instructions. This cell-permeant dye is non-fluorescent in a reduced state and exhibits bright orange fluorescence upon oxidation. CellROX Orange was chosen for measuring extracellular as well as intracellular ROS because this dye is compatible with full medium conditions and requires no cellular activation. For the detection of extracellular ROS, CellROX Orange was diluted to a final concentration of 10 μM in full cell culture medium, and IAE and test compounds were added as indicated in the corresponding results figure. Measurement was performed in black 96-well plates (#655090, Greiner Bio-One, Frickenhausen, Germany) in a final volume of 150 μl/well. To increase assay sensitivity, the dye was protected against atmospheric oxygen by adding a sealing layer of 100 μl mineral oil (Luxcel Biosciences, Cork, Ireland) to each well. Fluorescence intensity (529/590 nm) was recorded for 24 h at 37°C using the POLARstar Omega (BMG Labtech). Fluorescence values at time zero were subtracted for data analyses using GraphPad Prism 5.0. For the detection of intracellular ROS, HT-29 cells were seeded in 12-well plates (TPP, Biochrom) with a density of 250,000 cells/well one day before treatment. After treatment with IAE, MD or vehicle, cells were trypsinized and then washed with PBS. Pelleted cells were resuspended in 1 ml of 5 μM CellROX Orange in PBS and incubated for 20 min at 37°C. To remove unincorporated dye the cells were washed and resuspended again in PBS before flow cytometry detection (using an Accuri C6 device). Data were analyzed using FlowJo 7.6 (Tree Star) and GraphPad Prism 5.0.

### Detection of lipid peroxidation

Formation of cellular lipid peroxides was investigated by using the Click-iT Lipid Peroxidation assay (Life Technologies). This assay makes use of linoleamide alkyne (LAA, alkyne-modified linoleic acid) that assemble with cellular membranes. LAA can be oxidized resulting in alkyne-modified proteins that can be labeled using an azide-modified Alexa Fluor 488 dye through copper-catalyzed click chemistry. Thus, increasing fluorescence intensities are detected owing to enhanced lipid peroxidation upon treatment. One day before treatment HT-29 cells were seeded in 12-well plates with a density of 250,000 cells/well. Cells were then treated for 24 h with the IAE or menadione in presence of 50 μM LAA. After trypsinization, cells were washed with PBS and fixed in 3.7% formaldehyde for 15 min at room temperature. Cells were washed in PBS again, permeabilized by using 0.5% Triton X-100 in PBS for 10 min at room temperature and subsequently blocked by adding 1% BSA for 30 min at room temperature. Remaining BSA was removed by rigorously washing the cells with PBS. Pelleted cells were incubated with 500 μl Click-iT reaction cocktail for 30 min at room temperature according to the manufacturer’s protocol. The Click reaction was stopped by adding 1% BSA/PBS. The cells were again washed and resuspended with PBS. Flow cytometry was performed using the Accuri C6 device. Data were analyzed using FlowJo 7.6 (Tree Star) and GraphPad Prism 5.0.

### Fluorescence microscopy

For visualization of lipid peroxidation using the Click-iT Lipid Peroxidation method HT-29 cells were seeded on 13 mm cover slips placed in 24 well plates with a density of 125,000 cells/well one day before treatment. Cells were treated for 24 h with the IAE or menadione in presence of 50 μM linoleamide alkyne (LAA). Afterwards, adherent cells were washed with PBS and fixed in 3.7% formaldehyde for 15 min at room temperature. Cells were washed in PBS, permeabilized by using 0.5% Triton X-100 in PBS (PBS-T) for 10 min at room temperature and blocked by adding 1% BSA for 30 min at room temperature. After washing with PBS, 250 μl Click-iT reaction cocktail was added according to the manufacturer’s protocol and the cells were incubated for 30 min at room temperature. Reactions were stopped by washing with 1% BSA in PBS, and cells were again washed with BSA-free PBS. Cover slips were finally counterstained with ProLong Gold Antifade Mountant solution (containing DAPI) and incubated for 24 h at room temperature. Fluorescence microscopy was carried out using an LSM700 instrument (Zeiss, Jena, Germany).

For the detection of cleaved PARP, HT-29 cells were seeded on 13 mm cover slips (Sarstedt, Nürnbrecht, Germany) and placed in 24 well plates (Nunc, Wiesbaden, Germany) with a density of 125,000 cells/well one day before treatment. Cells were treated for 24 h with the IAE or test compounds, washed with PBS, and finally fixed in 4% formaldehyde/PBS for 15 min. Cells were permeabilized in 0.3% Triton X-100/PBS (PBS-T) for additional 10 min at room temperature and subsequently blocked in 5% goat serum (Sigma Aldrich) in PBS-T (0.3%) for 60 min at room temperature. Afterwards, cells were incubated overnight at 4°C with a primary antibody against cleaved PARP (#5625, Cell Signaling Technology), which was diluted (1:200) in 1% BSA/PBS-T (0.3%). Cells were washed with PBS-T (0.3%) and labeled by anti-rabbit IgG (H+L), F(ab')2 fragment Alexa Fluor 488 Conjugate (#4409, Cell Signaling Technology) diluted (1:1,000) in 1% BSA/PBS-T (0.3%) for 1 h at room temperature.

F-actin was stained with Texas Red-X phalloidin (Life Technologies) for 30 min at room temperature. Finally, cover slips were counterstained using ProLong Gold Antifade Mountant solution (containing DAPI) and incubated for 24 h at room temperature. Fluorescence microscopy was carried out using an LSM700 device (Zeiss, Jena, Germany).

### Cytotoxicity assay

To examine the effect of IAE on cell-viability in colon cancer and primary cells, we applied the CytoTox-Glo Cytotoxicity assay (Promega, Mannheim, Germany), as described recently [11]. Therefore, HT-29, and CCD 841 CoN cells were seeded in black 384-well plates (#3712, Corning, Wiesbaden, Germany) with a density of 5000 cells/well in a final volume of 25 μl/well. Cells were treated for 24h with concentration series of IAE by adding 10 μl of a 3.5-fold stock concentration. After treatment luminescence was measured using the POLARstar Omega device (BMG Labtech). Viability was calculated by subtraction of dead cell luminescence from total luminescence values. Data were fitted using GraphPad Prism 5.0 according to equation: Y = Bottom + (Top-Bottom)/(1+10^((X-LogIC50)).

### Tumor xenograft study

To study the *in vivo* efficacy of IAE for treating human CRC, a tumor growth inhibition study was performed using the HT-29 tumor xenograft mouse model. The study was approved on August 13^th^ 2014 by the Charles River Discovery Research Services NC Animal Care and Use Committee and conducted by Charles River Discovery Research Services (Morrisville, NC, USA). Animal welfare of Charles River Discovery Research Services was approved by the US Office of Laboratory Animal Welfare on December 14^th^ 2011 for the time period between December 14 2011 until October 31^st^ 2015 (identification number A4358-01). The study was performed in accordance with Charles River Discovery Research Services NC and US Public Health Service policies and guidelines (animal study protocol number: #980702). Briefly, 36 female athymic nude (NCr nu/nu) mice were implanted with 5 million HT-29 cells subcutaneously in the flank. At the day of inoculation, the mice were randomly divided into two groups (n = 18), and orally gavaged bidaily for 4 weeks with 50 mg/kg IAE in deionized water or with vehicle only. Tumor volumes and body weight were monitored during the entire experiment by Charles River Discovery Research Services and documentation of data was sent to the authors of this article for data analysis. At the end of the treatment, tumors were collected, weighed, and stored at -80°C prior to further analyses. Blood was collected by terminal cardiac puncture under isoflurane anesthesia, and serum was analyzed by clinical chemistry testing at Charles River.

### Statistical analyses

Data are expressed as mean ± standard error of mean (SEM) if not otherwise denoted. Statistical tests were performed using GraphPad Prism 5.0. For comparison of two groups statistical significance was examined by unpaired two-tailed Student’s t-test if not otherwise denoted. For multiple comparisons data were analyzed by one-way ANOVA with subsequent Dunnett’s post test. A *p* value ≤ 0.05 was defined as statistically significant.

## Results

### IAE inhibited proliferation of cancer cells and induced G2/M cell cycle arrest

**Fig 1:**
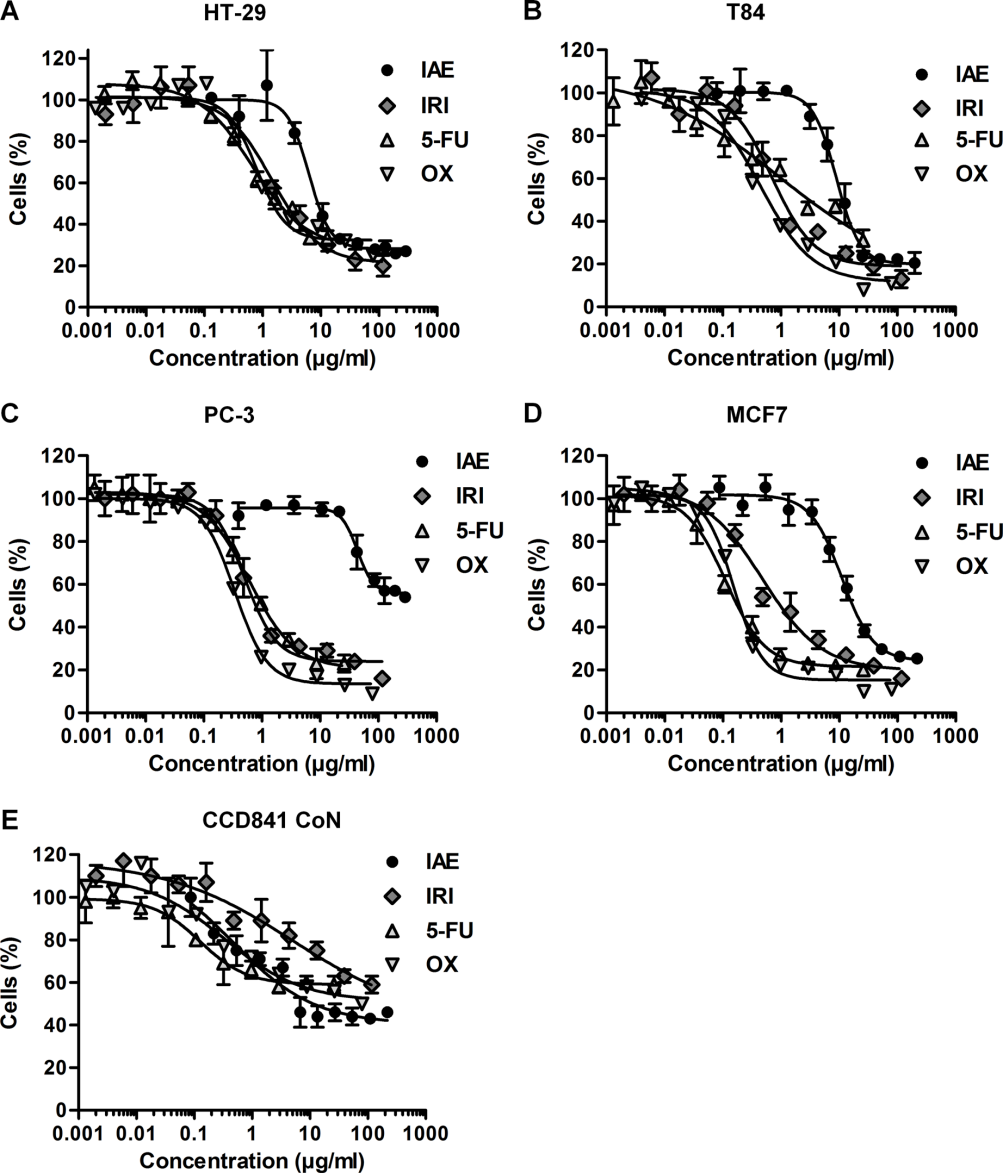
Inhibition of proliferation of HT-29 colon cancer cells (A), T84 colon cancer cells (B), PC-3 prostate cancer cells (C), MCF7 breast cancer cells (D) and normal CCD 841 CoN (colon) cells (E) after treatment with IAE and a small panel of anticancer drugs (namely irinotecan, 5-FU and oxiliplatin). Cells were treated with concentration series of IAE and compounds for 3 days (A and C) and 4 days (B and D, E), respectively. The relative number of cells was determined by measurement of cellular DNA content via fluorescent dye binding. Data are expressed as mean ± SD (n = 4).

To investigate the effects of IAE on the proliferation of colorectal cancer, HT-29 and T84 cells were treated with concentration series of IAE for 72 and 96 h (depending on the varying time required for cell proliferation of HT-29 and T84 cells), respectively. Cell proliferation was determined by measurement of cellular DNA content. IAE inhibited the proliferation of HT-29 and T84 colon carcinoma cells in a concentration-dependent manner with IC_50_ values (mean ± SD) of 6 ± 1 μg/ml (Fig 1A) and 9 ± 1 μg/ml (Fig 1B), respectively.

In the initial phase of the study, we additionally tested two unrelated cancer cell models, namely PC-3 (prostate) and MCF7 (breast), to roughly exclude cell specific effects of the IAE. Similarly to the two colon cancer models, IAE induced inhibition of proliferation (IC_50_ values of 44 ± 4 μg/ml (Fig 1C) in PC-3 and 11 ± 1 μg/ml (Fig 1D) in MCF7 cancer cells, respectively). These results suggested that the effects of IAE were not restricted to colon carcinoma cells.

As the cytotoxic anticancer drugs irinotecan, 5-fluorouracil (5-FU) and oxaliplatin, IAE showed treatment efficacies between 70 and 80%, indicating a fraction of 20–30% of still proliferating cancer cells after treatment (Fig 1, Table 1). In general, the IC_50_ value of IAE was about one order of magnitude higher compared to the applied compounds. Notably, in normal CCD 841 CoN cells (primary colon cells that derived from an infant), proliferation was inhibited by IAE—and also by 5-FU and oxaliplatin—at IC_50_ values that were about an order of magnitude lower than for the cancer cell lines (Fig 1E and Table 1). On the other hand, compared to cancer cells in primary colon cells inhibition efficiencies were clearly lower, indicating a higher number of still proliferating normal colon cells compared to the tested colon cancer cells.

**Table 1:** Effect of IAE and reference compounds on proliferation of HT-29 and T84 colon carcinoma cells, PC-3 prostate cancer, MCF7 breast cancer and normal CCD 841 CoN colon cells.

Cell lines	IC50 and Efficacy	IAE	Irinotecan	5-FU	Oxaliplatin
HT-29 (Colon cancer)	IC50 (μg/ml)	6.3 ± 0.9	1.5 ± 0.5	0.7 ± 0.1	0.8 ± 0.2
	Efficacy (%)	71.8 ± 2.1	78.7 ± 5.8	71.4 ± 5.0	67.7 ± 3.9
T-84 (Colon cancer)	IC50 (μg/ml)	9.0 ± 0.5	0.7 ± 0.2	0.9 ± 3.0	0.4 ± 0.1
	Efficacy (%)	79.9 ± 1.6	81.0 ± 4.8	86.2 ± 31.8	88.4 ± 3.9
PC3 (Prostate cancer)	IC50 (μg/ml)	43.8 ± 3.6	0.5 ± 0.1	0.6 ± 0.0	0.3 ± 0.0
	Efficacy (%)	44.2 ± 1.6	76.0 ± 2.3	79.4 ± 1.5	86.5 ± 1.6
MCF-7 (Breast cancer)	IC50 (μg/ml)	11.1 ± 1.1	0.5 ± 0.1	0.1 ± 0.0	0.2 ± 0.0
	Efficacy (%)	75.7 ± 2.7	80.2 ± 3.8	78.4 ± 1.6	84.7 ± 2.1
CCD 841 CoN (Colon normal)	IC50 (μg/ml)	0.5 ± 2.3	5.0 ± 19.2	0.1 ± 0.0	0.3 ± 0.3
	Efficacy (%)	59.6 ± 6.7	58.0 ± 24.7	41.0 ± 1.4	48.9 ± 6.4

Efficiency is the maximal observed induction of cell death after treatment relative to nontreated cells (set to 0%).

**Fig 2:**
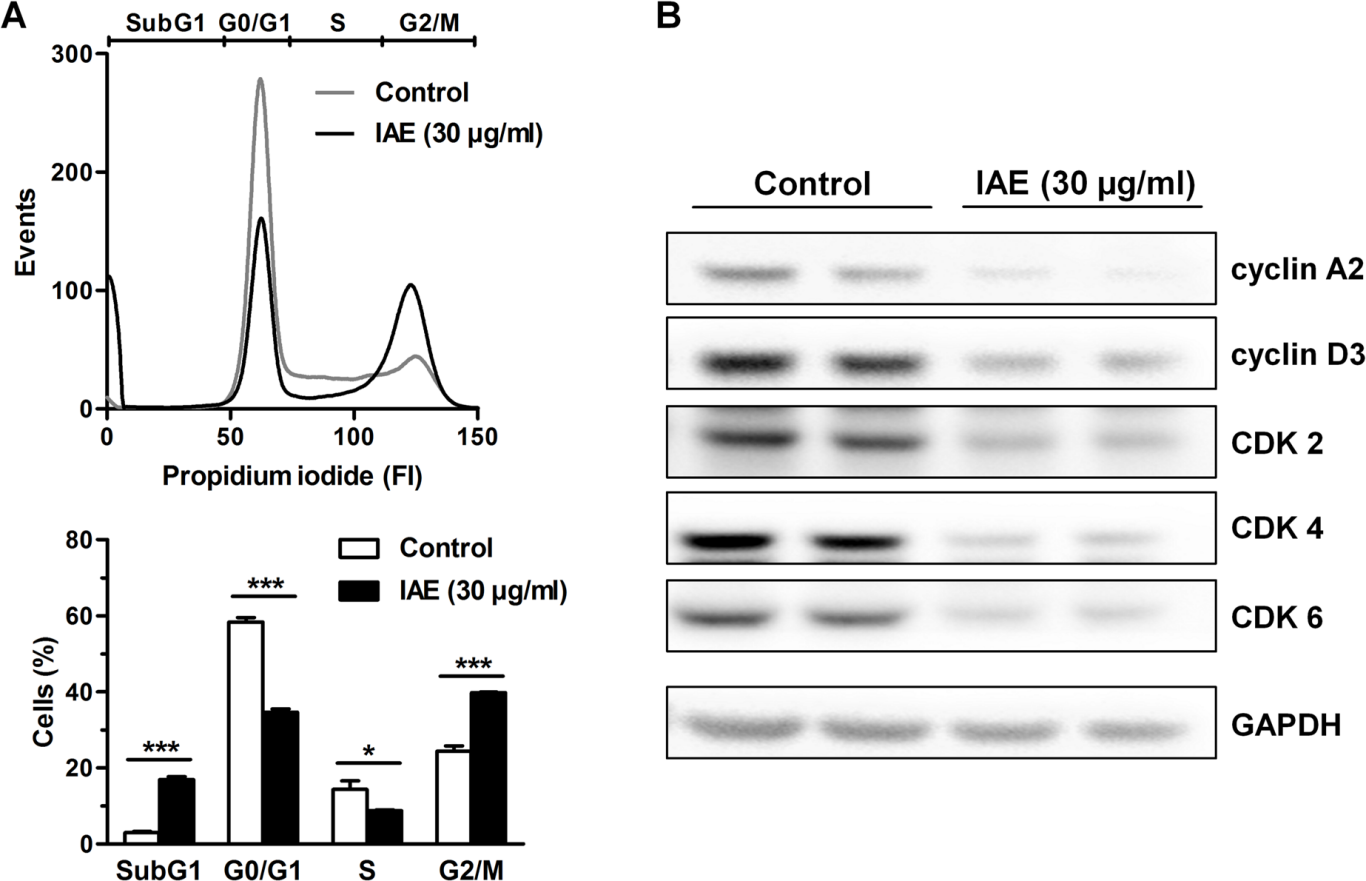
Cell cycle analysis of HT-29 colon carcinoma cells after treatment with 30 μg/ml IAE for 24 h. A, Cell cycle was analyzed by flow cytometry of propidium iodide stained cells. Histograms (top) show one representative experiment for each treatment condition. Bar plots (bottom) show percent of cell population in apoptotic SubG1, G0/G1, S and G2/M phases of the cell cycle and are expressed as mean ± SEM (n = 3). *p≤0.05, ***p≤0.001 vs. control. B, Whole cell lysates were analyzed for the expression of cyclin A2, cyclin D3, CDK2, CDK4, CDK6 and GAPDH proteins by immunoblotting using specific antibodies.

Cellular proliferation is generally linked to the progression of the cell cycle [12]. We thus asked if the observed antiproliferative effects of IAE are a result of changes in cell cycle regulation. We treated HT-29 colon carcinoma cells with 30 μg/ml IAE for 24 h and analyzed the cells after propidium iodide staining by flow cytometry. IAE treatment resulted in significant accumulation in the G2/M phase (40 vs. 24%) with concomitant reduction in the G0/G1 (35 vs. 58%) and the S phase (9 vs. 14%, Fig 2A). IAE further increased the number of cells in the SubG1 phase (17 vs 3%), indicating induction of apoptosis in these cells.

Furthermore, we analyzed the expression of cell cycle regulatory proteins by immunoblotting. Consistent with the flow cytometry data, IAE treatment reduced the expression of the cyclins A2 and D3, and of the cyclin dependent kinases (CDK) 2, 4, 6 (Fig 2B). Overall, the observed changes in expression of cell cycle regulatory proteins as well as the observed G2/M arrest possibly account for the cell growth inhibitory effects of IAE in colorectal cancer cells.

### IAE treatment activated caspases, induced DNA fragmentation and resulted in phosphatidylserine externalization in colon carcinoma cells

**Fig 3:**
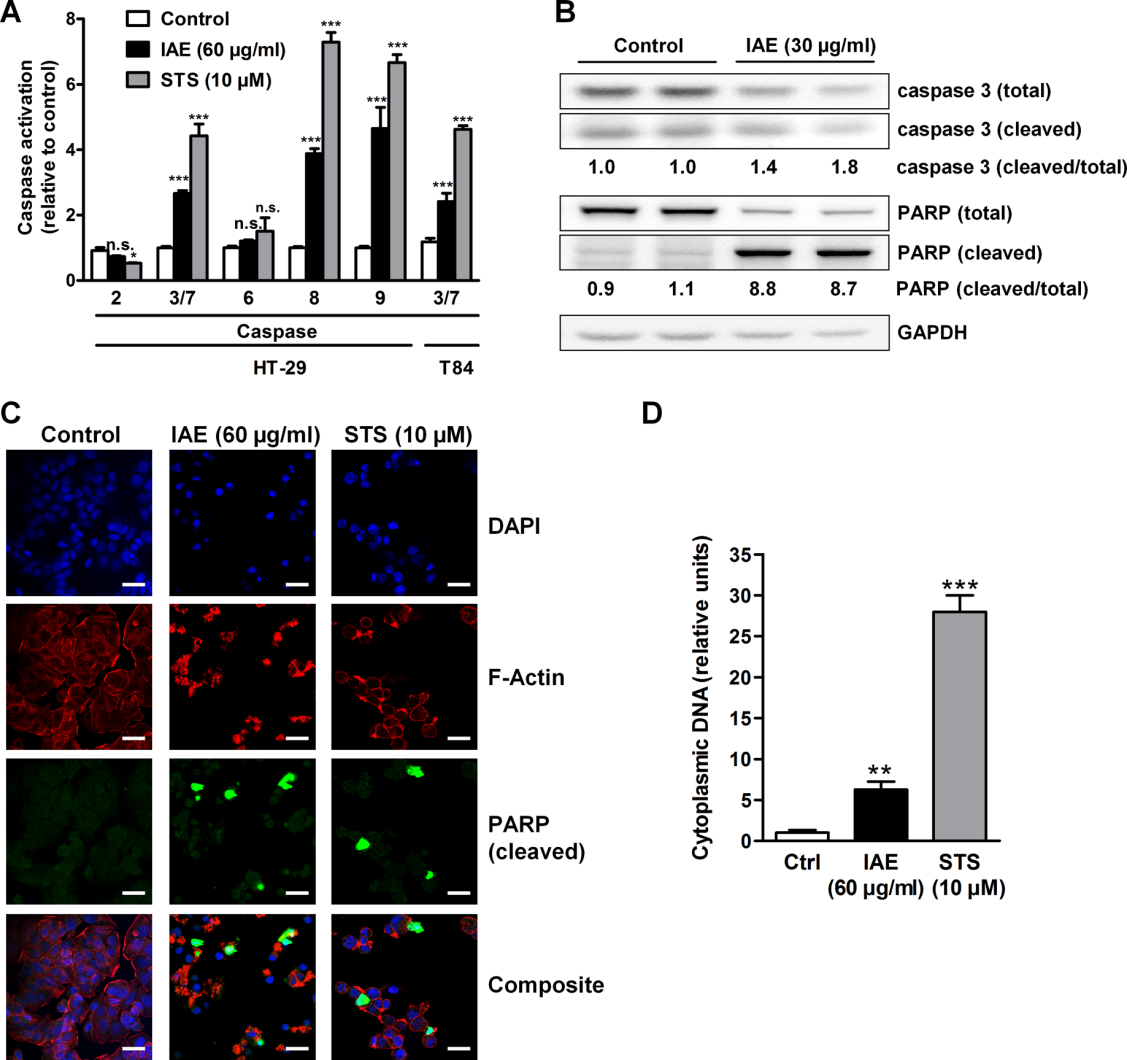
Activation of apoptosis signaling pathway in colon carcinoma cells after treatment with IAE. A, HT-29 and T84 cells were treated for 24 h. Enzymatic activation of caspases 2, 3/7, 6, 8 and 9 was determined by use of luminescence-based assays. Data are normalized to control treatment and are expressed as mean ± SEM (n = 4). B, Whole cell lysates from HT-29 cells treated for 24 h were analyzed for the expression of total and cleaved proteins of caspase 3, caspase 9 and PARP by immunoblotting. Numbers indicate densitometric ratios of the cleaved to total proteins normalized to control treatments. C, Fluorescence microscopy of HT-29 cells treated for 24 h. Cleaved caspase 3 was labeled green, F-actin red and the nucleus blue. Scale bars, 25 μm. D, HT-29 cells were treated for 6 h. DNA fragmentation was detected through accumulation of cytoplasmic BrdU-labeled DNA by ELISA. Data are normalized to control treatment and are expressed as mean ± SEM (n = 5). n.s. not significant, *p≤0.05, **p≤0.01, ***p≤0.001 vs. control.

Activation of the caspase signaling network is a hallmark of apoptosis, connecting extrinsic and intrinsic stimuli with downstream apoptotic events. In order to shed more light on the effects of IAE on the cellular caspase signaling network, we measured enzymatic activation of a panel of caspases using luminometric assays. Treatment of HT-29 cells with 60 μg/ml IAE for 24 h showed no effects on caspases 2 and 6. However, in HT-29 cells IAE treatment resulted in a significant 3-fold increase of the activity of the effector caspases 3/7, similar to application of 10 μM staurosporine (STN, 4-fold increase). Similar effects could be observed using an alternative (metastatic) colon cancer model (T84 cells), corroborating the results derived from the HT-29 cell model (Fig 3A).

Of note, IAE treatment increased the activity of both, caspase 8 and caspase 9, about 4-fold (Fig 3A), indicating induction of the extrinsic (death receptor mediated), as well as the intrinsic (mitochondrial mediated) pathway of apoptosis in HT-29 cells. As detected by immunoblotting, we additionally observed induced cleavage of the effector caspase 3, thus corroborating above-detected activation. IAE treatment further resulted in about 9-fold increased cleavage of the chromatin-associated poly (ADP-ribose) polymerase (PARP) (Fig 3B). PARP cleavage was also validated by fluorescence microscopy (Fig 3C). Since PARP is known to be activated upon DNA damage, we further investigated the effects of IAE on DNA integrity. In line with PARP cleavage, BrdU-labeling experiments showed elevated levels of cytosolic DNA in HT-29 cells treated with 60 μg/ml IAE for 6 h, indicating apoptotic DNA fragmentation upon IAE treatment (Fig 3D).

**Fig 4:**
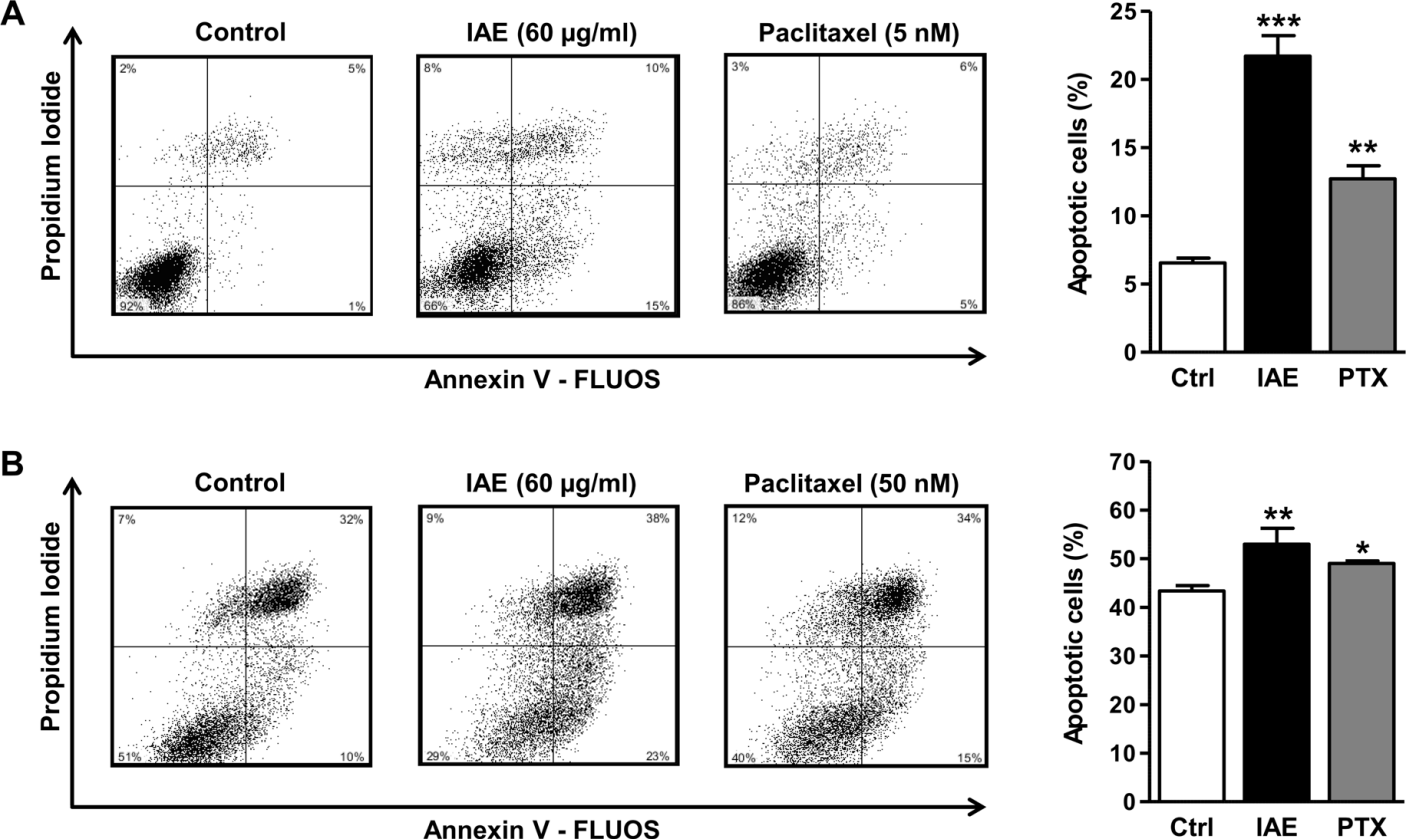
Annexin V assays of HT-29 (A) and T84 (B) colon carcinoma cells after treatment with IAE for 48 h. Apoptosis was determined by flow cytometry of annexin V-FLUOS and propidium iodide stained cells. Scatter plots show one representative experiment for each treatment condition. Bar plots indicate the percentage of apoptotic cells, comprising early (annexin positive, PI negative) and late stage (annexin positive, PI positive) events. Bars represent mean ± SEM (n = 3). *p≤0.05, **p≤0.01, ***p≤0.001 vs. control.

In healthy cells phosphatidylserine (PS) is generally restricted to the inner leaflet of the cell membrane, and exposure of phosphatidylserine on the outer leaflet is considered as a hallmark of apoptosis [13]. We thus treated colon carcinoma cells with 60 μg/ml IAE for 48 h and determined PS externalization by flow cytometry of annexin V-FLUOS/propidium iodide-labeled cells. IAE significantly increased the number of apoptotic cells from 6% to 22% in HT-29 (Fig 4A) and from 43% to 53% in T84 cells (Fig 4B). Similar observations were made with low doses of the anticancer drug paclitaxel (5–50 nM) (Fig 4). Overall, these data reveal that IAE efficiently induced apoptosis in human colon cancer cells.

**Fig 5:**
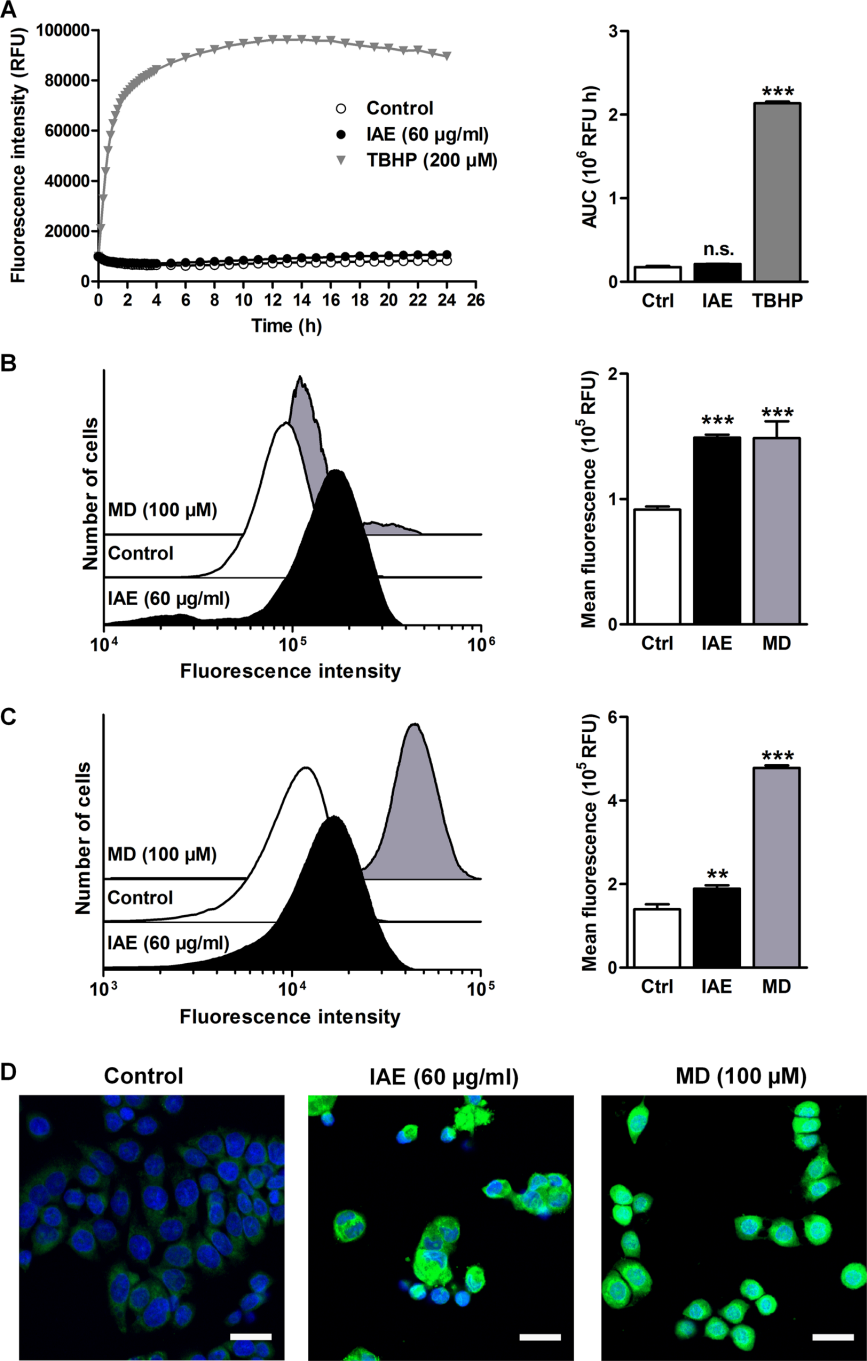
Extra- and intracellular formation of reactive oxygen species (ROS) and of lipid peroxides in HT-29 cells after treatment with IAE. A, Extracellular formation of ROS in full cell culture medium was kinetically detected using the ROS-sensitive CellROX Orange fluorogenic probe. Data are expressed as mean ± SEM (n = 8). B, Intracellular ROS was detected by flow cytometry of HT-29 cells stained with CellROX Orange after treatment for 24 h. Histograms (left) show one representative experiment for each treatment condition. Bar plots (right) show fluorescence intensities as mean ± SEM (n = 6). C, Intracellular lipid peroxidation was detected by flow cytometry of cells treated for 24 h using the Click-iT technology. Increasing fluorescence intensities are a result of enhanced lipid peroxidation upon treatment. Histograms (left) show one representative experiment for each treatment condition. Bar plots (right) show fluorescence intensities as mean ± SEM (n = 6). D, Intracellular lipid peroxidation was visualized by fluorescence microscopy (green, lipid peroxides; blue, nucleus). Scale bars, 25 μm. n.s. not significant, **p≤0.01, ***p≤0.001 vs. control.

Remarkably, cancer cells feature an imbalance of reactive oxygen species (ROS). Since elevated concentrations of ROS can induce apoptosis, increasing oxidative stress to cancer cells could be a promising strategy for therapy [14]. To investigate the potential role of ROS during IAE treatment, we first determined the formation of extracellular ROS. For that purpose, we incubated full cell culture medium with 60 μg/ml IAE at 37°C and detected generation of ROS within 24 h using the CellROX Orange fluorogenic probe. Noteworthy, in contrast to tert-butyl hydroperoxide (TBHP), IAE induced no detectable extracellular ROS (Fig 5A). However, IAE considerably increased intracellular ROS levels similar to the free radical generator menadione (MD, Fig 5B), as could be detected by using the same (cell-permeant) fluorogenic probe.

Since the formation of ROS can lead to increased intracellular oxidation of lipids we further investigated the effects of IAE treatment on lipid peroxidation in these cells. IAE considerably induced lipid peroxidation (Fig 5C and 5D), which provides evidence that IAE treatment could result in formation of ROS and resulting growth arrest and apoptosis in HT-29 colon carcinoma cells.

**Fig 6:**
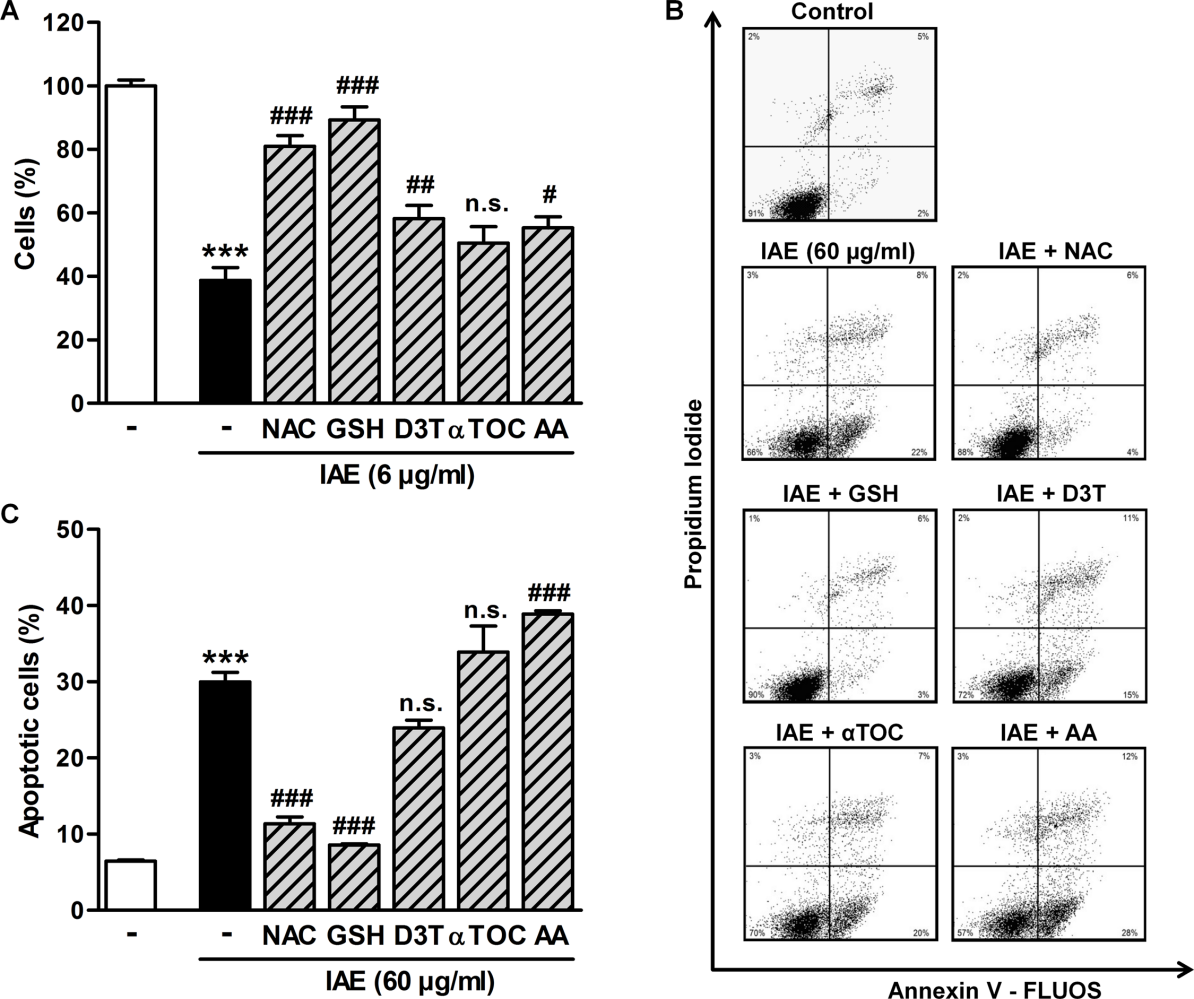
Effects of IAE on inhibition of proliferation (A) and apoptosis (B, C) during co-treatment with antioxidants. A, HT-29 colon carcinoma cells were treated with 6 μg/ml IAE for 48 h in presence or absence of the antioxidants NAC (1 mM), GSH (2.5 mM), D3T (25 μM), αTOC (50 μM) and AA (0.5 mM), respectively. The relative number of cells was determined by measuring cellular DNA content via fluorescent dye binding. Data are expressed as mean ± SD (n = 4). B, HT-29 cells were treated with 60 μg/ml IAE for 24 h in presence or absence of the antioxidants NAC (1 mM), GSH (5 mM), D3T (50 μM), αTOC (50 μM) and AA (1 mM), respectively. Apoptosis was determined by flow cytometry of annexin V-FLUOS and propidium iodide stained cells. Scatter plots show one representative experiment for each treatment condition. C, Bar plots of annexin flow cytometry data (B) indicate the fraction of apoptotic cells, comprising early (annexin positive, PI negative) and late stage (annexin positive, PI positive) events. Bars represent mean ± SEM (n = 4). ***p≤0.001 vs. untreated cells (control); #p≤0.05, ##p≤0.01, ###p≤0.001 vs. IAE-only treated cells. n.s. not significant vs. IAE-only treated cells.

We next analyzed the role of ROS in IAE-induced inhibition of proliferation and apoptosis. Therefor, we co-treated HT-29 cells with IAE and antioxidants and measured—as described above—markers for proliferation and apoptosis. Strikingly, N-acetyl cysteine (NAC) and glutathione (GSH) almost completely prevented the antiproliferative effects of 6 μg/ml IAE after 48 h of treatment (Fig 6A), whereas application of 3H-1, 2-dithiole-3-thione (D3T), α-tocopherol (αTOC) and ascorbic acid (AA) was less efficient (Fig 6A). Remarkably, annexin V based assays indicated that NAC and GSH almost completely protected HT-29 cells from apoptosis, which was induced by treatment with 60 μg/ml IAE for 24 h (Fig 6B and 6C). In summary, our mechanistic analyses suggest that intracellular formation of ROS contributes significantly to cell death in IAE-treated colon carcinoma cells *in vitro*.

### IAE inhibits tumor growth in mice

**Fig 7:**
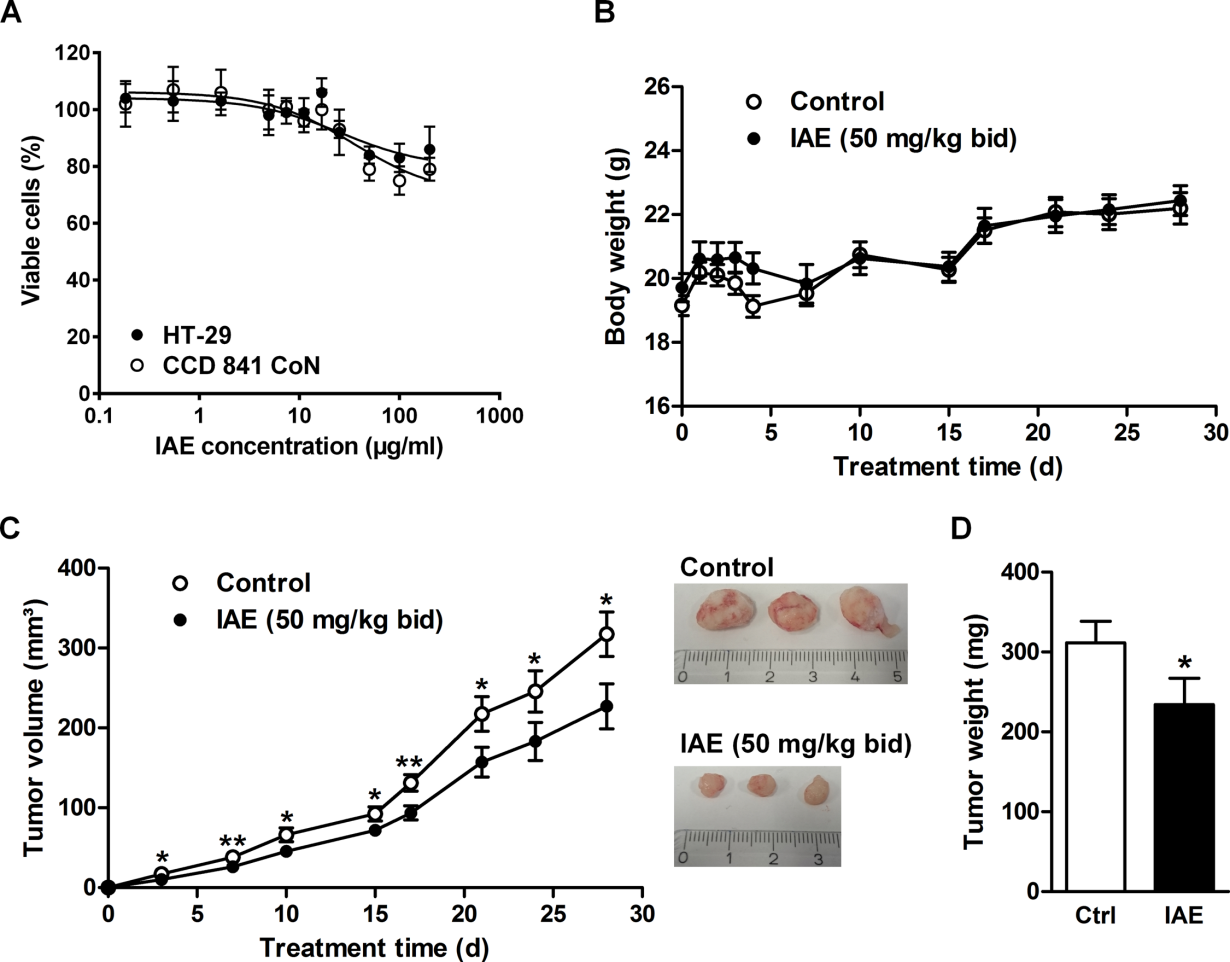
Cytotoxicity test and evaluation of inhibition of tumor growth by treating HT-29 tumor xenograft mouse models with IAE. Athymic nude mice were ectopically implanted with 5 million HT-29 cells in the flank and orally gavaged bidaily by 50 mg/kg IAE or vehicle for 4 weeks. A, Cytotoxicity was tested for colon cancer and primary colon cells (IC_50_ = 25.82 μg/mL (HT-29); IC_50_ = 36.12 μg/mL (CCD 841 CoN)). Data are expressed as mean ± SD (n = 4). B, Body weight during entire experiment. C, Tumor volume during entire experiment and images of exemplary xenografts of untreated and IAE-treated mice at end point. D, Tumor weight at the end of the study. Data are expressed as mean ± SEM (n = 18). n.s. not significant, *p≤0.05, **p≤0.01, ***p≤0.001 vs. control (one-tailed t test).

The here studied IAE is used as a safe, clinically validated product that has been applied for many years by millions of patients. Nevertheless, as shown in Fig 1 and Table 1, IAE inhibited proliferation not only in cancer but with an even lower IC_50_ value in normal colon cells (although with less efficiency). Before starting with an animal experiment we additionally tested the IAE for potential adverse effects on cell viability. IAE showed by trend slightly higher efficiency for tumor versus primary cells (Fig 7A). Notably, almost 80% of the overall cell population (of which in general only a fraction is proliferating) remained viable, indicating potentially mild effects for application *in vivo*.

We finally asked if IAE treatment could potentially generate anticancerogenic effects *in vivo* using the well-established HT-29 colon cancer xenograft mouse model. We analyzed potential inhibition of tumor growth after bidaily oral application of a conventional phytotherapeutic dose of 50 mg/kg IAE over 4 weeks. Relative to vehicle-treated control mice, IAE-treated mice maintained constant body weight (Fig 7B) and showed unaltered liver function parameters (Table 2), suggesting lack of relevant toxic side effects, which is in line with the above-described cell viability experiments. Strikingly, after few days we observed significant inhibition of tumor growth as a result of IAE treatment (Fig 7C). After 4 weeks tumor weight was even reduced by 25% in IAE-treated mice relative to control mice (Fig 7D). Higher concentrations than 50 mg/kg of IAE did not improve this parameter (data not shown). In summary, the overall safe IAE showed moderate but significant antitumor effects *in vivo*.

**Table 2:** Liver and kidney panel assays at the end of the 4-weeks tumor growth inhibition study.

Serum Parameter	Control	IAE (50 mg/kg bid)	*p* value
Protein (g/dl)	4.07 ± 0.07	3.99 ± 0.09	0.504
Albumin (g/dl)	2.39 ± 0.04	2.37 ± 0.05	0.723
Globulin (g/dl)	1.67 ± 0.06	1.62 ± 0.07	0.555
Albumin/Globulin ratio	1.46 ± 0.06	1.51 ± 0.07	0.543
AST (U/l)	217 ± 69	149 ± 31	0.381
ALT (U/l)	51 ± 7	42 ± 3	0.199
Alkaline Phosphatase (U/l)	64 ± 4	74 ± 3	0.056
Bilirubin (mg/dl)	0.16 ± 0.01	0.15 ± 0.01	0.590
Urea Nitrogen (mg/dl)	20 ± 1	19 ± 1	0.280

AST, aspartate aminotransferase; ALT, alanine aminotransferase; bid, bidaily.

## Discussion

Recently, it was reported that IAE potentially exerts antiproliferative effects in HT-29 *in vitro*, but underlying mechanisms of action or further details were not shown [15]. Here, we demonstrated for the first time in which way hydroethanolic extracts of *Iberis amara* totalis might inhibit the proliferation of human colon cancer cells.

IAE induced cell cycle arrest and apoptosis in colon carcinoma cells *in vitro*. Notably, we identified intracellular formation of ROS and lipid peroxidation of membranes the main mechanisms to kill tumor cells such as HT-29, whereas quenching of ROS by antioxidants rescued cancer cells from IAE-induced growth inhibition and apoptosis [14].

To our knowledge, intracellular formation of ROS has never been reported for IAE so far. Previous studies showed radical scavenging and reducing properties of IAE, predominantly in cell-free systems [16, 17]. The role of redox-active molecules in balancing the redox state is controversially discussed, especially for phenols, flavonoids and other natural products [18–21]. Generally, these redox-active molecules can possess opposing properties. As antioxidative agents these molecules tend to scavenge free radicals. But depending on environmental conditions, redox-active molecules can on the contrary pro-oxidatively trigger the formation of ROS (e.g. hydrogen peroxide, hydroxyl radicals) [22]. The balance between anti- and pro-oxidative activities of redox-active molecules depends in general on molecular conditions such as: i) the concentration of the redox-active molecule; ii) the presence of other pro-/antioxidants; iii) the solvent used; iv) the microoxygen environment (e.g. partial pressure of oxygen), and v) the concentration of metal ions [19]. IAE contains a large pool of natural products, including glucosinolates, fatty acids, phenolic acids, flavonoids and triterpenes [10]. These compounds can disturb ROS balance and eventually exert antiproliferative and apoptotic effects *in vitro* [23–26]. Moreover, IAE might interfere with mitochondrial function to produce cellular stress resulting in elevated endogenous production of ROS.

Health-beneficial effects of plant extracts are known as an emerging property deriving from synergistic or at least additive effects of pools of natural products—the sum of the whole is more than its single entities. Isolation of “the active ingredient” from the whole extract is thus considered not useful. Moreover, pharmacological *in vivo* studies that are conventionally applied for single compounds including pharmacokinetics or pharmacodynamics analyses can obviously not be performed with complex compound pools of extracts. These typical features of phytomedical research make it difficult to convey observed effects with similar scientific rigor and mechanistic simplicity known from analyses of single compounds.

Nevertheless, induction of cellular ROS was observed *in vitro*, which might explain at least in part the here observed significant tumor-growth inhibition *in vivo*. In contrast to the HT-29 cell model, HT-29-xenografts of IAE-treated mice showed varying, non-significant trends of markers for proliferation or apoptosis (data not shown). The modest comparability of molecular markers between *in vitro* and *in vivo* models might be explained by unequal metabolization of compounds pools, different treatment conditions *in vitro* and *in vivo* (e.g. treatment time and dose) and varying microenvironments of cultured cells and xenografts. Notably, it remains difficult to estimate the potential interferences of IAE with diet. Excessive consumption of antioxidants could potentially preclude beneficial effects of low amounts of here observed endogenously produced ROS [27]. Future studies might address the complex interference of supplementation of antioxidants and ROS-inducing cancer therapeutics in more detail.

This study focused on the discovery of potential anticancerogenic effects of a generally well-accepted safe phytoextract. In general, we applied a common panel of assays for characterizing cytotoxic events. However, the mode of action of cytotoxic cancer chemotherapy drugs is still not fully understood, and conventional mechanistic experiments that are performed *in vitro* are quite different from the much more complex situation *in vi*vo [28]. For example, median proliferation rates of cells are usually much higher in cell culture than in tissues. Selectivity against proliferating cells is believed as an efficient strategy for treating cancer. But proliferation properties are usually low in most chemosensitive cancers, making it difficult to compare treatments *in vitro* and *in vivo*, as evidenced by mentioned difficulties to detect biomarkers *in vivo*. Different proliferation rates of cancerogenic and normal cells shall also be considered when making a simple comparison of proliferation curves (Fig 1A–1E, Table 1). Notably, not only the IAE but also the cytotoxic colon cancer drugs 5-FU and oxaliplatin show indeed antiproliferative effects at lower doses in normal compared to cancerogenic cells; nevertheless, although not optimal due to (cytotoxic) side effects on normal tissues, these drugs can help a lot to cure colon cancer.

Timothy J. Mitchison suggested four potential but still largely unexplored explanations for the so-called “proliferation rate paradoxon” of cancer treatments [28], which may also be helpful to better understand the mode of action of IAE, namely:

1. Drug retention in tumors, meaning that drugs or extracts such as IAE would remain in tumor tissue cells much longer than in the circulation. Further extensive analytical experiments would be required to detect the life-time of compounds derived from IAE in the tumor and the circulation system, and to explore specific properties of compounds or compound mixtures in a tumor microenvironment. However, this effort would exceed current analytical standards, in particular for the analysis of complex phytoextracts.
2. Targeting non-cancer cells within the tumor. As a result of potential retention of IAE in the tumor, cytotoxic effects on other tumor-infiltrating (immune) cells might play a role, which is difficult to analyze and would require further extensive studies (e.g. molecular dissection of cell populations of tumors).
3. Bystander killing. IAE might initially only target a subset of cancer cells, but these damaged cells might affect neighboring tumor cells to induce an avalanche of cancer-killing reactions including various cell types within the tumor. Even mild and hardly detectable initial molecular events might thus trigger apoptotic effects in tumors.
4. Killing of quiescent cancer cells. As shown, IAE treatment resulted in increasing levels of ROS leading to enhanced rates of apoptosis of cancer cells.

In summary, although some assays might hint to even higher cytotoxic effects in normal versus cancer cells, the pharmacological response of compounds or extracts can differ significantly in an *in vivo* context. Further basic research is warranted to better understand the principles of cytotoxic anticancer drugs, thus providing a more rational frame to test new cancer therapeutics. Moreover, additional cell line models (e.g. considering Duke types A-D) might help to validate the here-observed anticancerogenic actions but on the other hand such cell line models can still vary a lot from real tumors (e.g. due to varying genetic background).

Interestingly, for the here applied IAE gastrointestinal human health claims have been officially approved. and this extract has shown good safety profiles in various toxicity, genotoxicity and mutagenicity analyses [10], consisted with our mouse model study.

Amongst others, IAE may thus become a useful phytomedical alternative to support cytotoxic or targeted (e.g. antibody-based) drug treatments, or application of IAE might become helpful to inhibit tumor relapse after successful medication. Further in-depth and clinical human studies are clearly necessary to evaluate the anti-cancerogenic potential of IAE, including for example additional preclinical studies for figuring out the optimal dosage for application in humans. Since IAE is widely accepted for alleviation of gastrointestinal disorders, its potential utility for the prevention and (co-) treatment of gastrointestinal cancers and cancer recurrences seems promising.

## References

[1] Brenner H, Kloor M, Pox CP. Colorectal cancer. Lancet. 2014;383(9927):1490–502. 10.1016/S0140-6736(13)61649-9 . 24225001

[2] Shekhar MP. Drug resistance: challenges to effective therapy. Curr Cancer Drug Targets. 2011;11(5):613–23. . 21486215 10.2174/156800911795655921

[3] Braun MS, Seymour MT. Balancing the efficacy and toxicity of chemotherapy in colorectal cancer. Ther Adv Med Oncol. 2011;3(1):43–52. 10.1177/1758834010388342 21789155 PMC3126034

[4] Ko JK, Auyeung KK. Target-oriented mechanisms of novel herbal therapeutics in the chemotherapy of gastrointestinal cancer and inflammation. Curr Pharm Des. 2013;19(1):48–66. . 22950499 10.2174/13816128130109

[5] Wagner H, Ulrich-Merzenich G. Synergy research: approaching a new generation of phytopharmaceuticals. Phytomedicine. 2009;16(2–3):97–110. 10.1016/j.phymed.2008.12.018 . 19211237

[6] Weidner C, Wowro SJ, Rousseau M, Freiwald A, Kodelja V, Abdel-Aziz H, et al Antidiabetic Effects of Chamomile Flowers Extract in Obese Mice through Transcriptional Stimulation of Nutrient Sensors of the Peroxisome Proliferator-Activated Receptor (PPAR) Family. PloS ONE. 2013;8(11):e80335 10.1371/journal.pone.0080335 24265809 PMC3827197

[7] Weidner C, Rousseau M, Plauth A, Wowro SJ, Fischer C, Abdel-Aziz H, et al Melissa officinalis extract induces apoptosis and inhibits proliferation in colon cancer cells through formation of reactive oxygen species. Phytomedicine. 2015;22(2):262–70. 10.1016/j.phymed.2014.12.008 . 25765831

[8] Weidner C, Wowro SJ, Freiwald A, Kodelja V, Abdel-Aziz H, Kelber O, et al Lemon balm extract causes potent antihyperglycemic and antihyperlipidemic effects in insulin-resistant obese mice. Mol Nutr Food Res. 2014;58(4):903–7. 10.1002/mnfr.201300477 . 24272914

[9] Ammon HP, Kelber O, Okpanyi SN. Spasmolytic and tonic effect of Iberogast (STW 5) in intestinal smooth muscle. Phytomedicine. 2006;13 Suppl 5:67–74. 10.1016/j.phymed.2006.08.004 . 16978852

[10] Kroll U, Cordes C. Pharmaceutical prerequisites for a multi-target therapy. Phytomedicine. 2006;13 Suppl 5:12–9. Epub 2006/07/22. doi: S0944-7113(06)00080-8 [pii] 10.1016/j.phymed.2006.03.016 . 16857355

[11] Fuhr L, Rousseau M, Plauth A, Schroeder FC, Sauer S. Amorfrutins Are Natural PPARgamma Agonists with Potent Anti-inflammatory Properties. Journal of natural products. 2015;78(5):1160–4. 10.1021/np500747y . 25938459

[12] Hartwell LH, Kastan MB. Cell cycle control and cancer. Science. 1994;266(5192):1821–8. . 7997877 10.1126/science.7997877

[13] Fadok VA, Bratton DL, Frasch SC, Warner ML, Henson PM. The role of phosphatidylserine in recognition of apoptotic cells by phagocytes. Cell Death Differ. 1998;5(7):551–62. 10.1038/sj.cdd.4400404 . 10200509

[14] Yang Y, Karakhanova S, Werner J, Bazhin AV. Reactive oxygen species in cancer biology and anticancer therapy. Curr Med Chem. 2013;20(30):3677–92. . 23862622 10.2174/0929867311320999165

[15] Bonaterra GA, Kelber O, Weiser D, Kinscherf R. Mechanisms of the anti-proliferative and anti-inflammatory effects of the herbal fixed combination STW 5 (Iberogast(R)) on colon adenocarcinoma (HT29) cells in vitro. Phytomedicine. 2013;20(8–9):691–8. 10.1016/j.phymed.2013.02.011 . 23535188

[16] Germann I, Hagelauer D, Kelber O, Vinson B, Laufer S, Weiser D, et al Antioxidative properties of the gastrointestinal phytopharmaceutical remedy STW 5 (Iberogast). Phytomedicine. 2006;13 Suppl 5:45–50. 10.1016/j.phymed.2006.03.018 . 16713223

[17] Schempp H, Weiser D, Kelber O, Elstner EF. Radical scavenging and anti-inflammatory properties of STW 5 (Iberogast) and its components. Phytomedicine. 2006;13 Suppl 5:36–44. 10.1016/j.phymed.2006.03.017 . 16777393

[18] Halliwell B. Are polyphenols antioxidants or pro-oxidants? What do we learn from cell culture and in vivo studies? Arch Biochem Biophys. 2008;476(2):107–12. 10.1016/j.abb.2008.01.028 . 18284912

[19] Bouayed J, Bohn T. Exogenous antioxidants—Double-edged swords in cellular redox state: Health beneficial effects at physiologic doses versus deleterious effects at high doses. Oxid Med Cell Longev. 2010;3(4):228–37. 10.4161/oxim.3.4.12858 20972369 PMC2952083

[20] Khan HY, Zubair H, Ullah MF, Ahmad A, Hadi SM. A prooxidant mechanism for the anticancer and chemopreventive properties of plant polyphenols. Curr Drug Targets. 2012;13(14):1738–49. . 23140285 10.2174/138945012804545560

[21] Quideau S, Deffieux D, Douat-Casassus C, Pouysegu L. Plant polyphenols: chemical properties, biological activities, and synthesis. Angew Chem Int Ed Engl. 2011;50(3):586–621. 10.1002/anie.201000044 . 21226137

[22] Forester SC, Lambert JD. The role of antioxidant versus pro-oxidant effects of green tea polyphenols in cancer prevention. Mol Nutr Food Res. 2011;55(6):844–54. 10.1002/mnfr.201000641 21538850 PMC3679539

[23] Hayes JD, Kelleher MO, Eggleston IM. The cancer chemopreventive actions of phytochemicals derived from glucosinolates. Eur J Nutr. 2008;47 Suppl 2:73–88. 10.1007/s00394-008-2009-8 . 18458837

[24] Dinkova-Kostova AT, Kostov RV. Glucosinolates and isothiocyanates in health and disease. Trends Mol Med. 2012;18(6):337–47. 10.1016/j.molmed.2012.04.003 . 22578879

[25] Kong Y, Chen J, Zhou Z, Xia H, Qiu MH, Chen C. Cucurbitacin E induces cell cycle G2/M phase arrest and apoptosis in triple negative breast cancer. PloS ONE. 2014;9(7):e103760 10.1371/journal.pone.0103760 25072848 PMC4114842

[26] Hoensch HP, Kirch W. Potential role of flavonoids in the prevention of intestinal neoplasia: a review of their mode of action and their clinical perspectives. Int J Gastrointest Cancer. 2005;35(3):187–95. 10.1385/IJGC:35:3:187 . 16110120

[27] Ristow M, Zarse K, Oberbach A, Kloting N, Birringer M, Kiehntopf M, et al Antioxidants prevent health-promoting effects of physical exercise in humans. Proc Natl Acad Sci U S A. 2009;106(21):8665–70. 10.1073/pnas.0903485106 19433800 PMC2680430

[28] Mitchison TJ. The proliferation rate paradox in antimitotic chemotherapy. Mol Biol Cell. 2012;23(1):1–6. 10.1091/mbc.E10-04-0335 22210845 PMC3248889

